# Clinical and prognostic value of tumor volumetric parameters in melanoma patients undergoing ^18^F-FDG-PET/CT: a comparison with serologic markers of tumor burden and inflammation

**DOI:** 10.1186/s40644-020-00322-1

**Published:** 2020-07-06

**Authors:** Christian Philipp Reinert, Sergios Gatidis, Julia Sekler, Helmut Dittmann, Christina Pfannenberg, Christian la Fougère, Konstantin Nikolaou, Andrea Forschner

**Affiliations:** 1 Department of Radiology, Diagnostic and Interventional Radiology, University Hospital Tübingen, Hoppe-Seyler-Str.3, 72076 Tübingen, Germany; 2 Department of Radiology, Nuclear Medicine and Clinical Molecular Imaging, University Hospital, Tübingen, Hoppe-Seyler-Str.3, 72076 Tübingen, Germany; 3 Cluster of Excellence iFIT (EXC 2180) “Image Guided and Functionally Instructed Tumor Therapies”, University of Tübingen, Tübingen, Germany; 4 German Cancer Consortium (DKTK). Partner Site Tübingen, Tübingen, Germany; 5 Department of Dermatology, University Hospital Tübingen, Liebermeisterstrasse 25, 72076 Tübingen, Germany

**Keywords:** Malignant melanoma, ^18^F-FDG-PET/CT, Tumor volumetric parameter, Overall survival, Biomarker

## Abstract

**Background:**

To investigate the association of tumor volumetric parameters in melanoma patients undergoing ^18^F-FDG-PET/CT with serologic tumor markers and inflammatory markers and the role as imaging predictors for overall survival.

**Methods:**

A patient cohort with advanced melanoma undergoing ^18^F-FDG-PET/CT for planning metastasectomy between 04/2013 and 01/2015 was retrospectively included. The volumetric PET parameters whole-body MTV and whole-body TLG as well as the standard uptake value (SUV) peak were quantified using 50%-isocontour volumes of interests (VOIs) and then correlated with the serologic parameters lactate dehydrogenase (LDH), S-100 protein, c-reactive protein (CRP) and alkaline phosphatase (AP). PET parameters were dichotomized by their respective medians and correlated with overall survival (OS) after PET/CT. OS was compared between patients with or without metastases and increased or not-increased serologic parameters.

**Results:**

One hundred seven patients (52 female; 65 ± 13.1yr.) were included. LDH was strongly associated with MTV (r_P_ = 0.73, *p* <  0.001) and TLG (r_P_ = 0.62, p <  0.001), and moderately associated with SUV_peak_ (r_P_ = 0.55, *p* <  0.001). S-100 protein showed a moderate association with MTV (r_P_ = 0.54, *p* <  0.001) and TLG (r_P_ = 0.48, p <  0.001) and a weak association with SUV_peak_ (r_P_ = 0.42, *p* <  0.001). A strong association was observed between CRP and MTV (r_P_ = 0.66, *p* <  0.001) and a moderate to weak association between CRP and TLG (r_P_ = 0.53, p <  0.001) and CRP and SUV_peak_ (r_P_ = 0.45, p <  0.001). For differentiation between patients with or without metastases, receiver operating characteristic (ROC) analysis revealed a cut-off value of 198 U/l for serum LDH (AUC 0.81, sensitivity 0.80, specificity 0.72).

Multivariate analysis for OS revealed that both MTV and TLG were strong independent prognostic factors. TLG, MTV and SUV_peak_ above patient median were accompanied with significantly reduced estimated OS compared to the PET parameters below patient median (e.g. TLG: 37.1 ± 3.2 months vs. 55.9 ± 2.5 months, *p* <  0.001). Correspondingly, both elevated serum LDH and S-100 protein were accompanied with significantly reduced OS (36.5 ± 4.9 months and 37.9 ± 4.4 months) compared to normal serum LDH (49.2 ± 2.4 months, *p* = 0.01) and normal S-100 protein (49.0 ± 2.5 months, p = 0.01).

**Conclusions:**

Tumor volumetric parameters in ^18^F-FDG-PET/CT serve as prognostic imaging biomarkers in patients with advanced melanoma which are associated with established serologic tumor markers and inflammatory markers.

## Background

Malignant melanoma incidence is increasing worldwide. At time of diagnosis, most patients have localized disease that can be successfully treated by complete surgical resection, however, 28% of stage IV melanoma patients develop visceral metastases [1]. Recently, new treatment approaches such as antibodies targeting the immune checkpoints T-lymphocyte-associated protein 4 (CTLA-4) or the programmed cell death protein 1 (PD-1) either used alone or as combined immunotherapy remarkably improved prognosis of advanced melanoma. However, about 40–50% of patients fail to respond to therapy [2–5].

Serum lactate dehydrogenase (LDH) is released through cell damage and has been established as a biochemical marker of tumor load in various tumor entities including malignant melanoma [6]. Serum LDH is part of the AJCC melanoma staging guideline for metastatic melanoma patients [6]. Elevated serum LDH level is associated with poor survival and poor therapy response rates [5, 7, 8].

The calcium-binding, acidic cytoplasmic S-100 protein has been shown to be a specific and reliable immunohistochemical marker in malignant melanoma which correlates with clinical melanoma stage and poor survival [9–13]. Besides, several studies have found that the inflammatory markers c-reactive protein (CRP) and alkaline phosphatase (AP) are independent prognostic biomarkers in patients with both early-stage and advanced-stage melanoma [14–16].

Whole-body ^18^F-FDG-PET/CT is the imaging modality of choice for staging of advanced (stage III and IV) melanoma to provide information on the presence and location of metastases [17]. For assessing the degree of ^18^F-FDG accumulation in diverse cancer types, the volumetric parameters MTV and TLG have been proposed, as they reflect the whole volume of the tumor rather than the maximum standardized uptake value (SUV_max_) which represents only the most active part of the tumor [18–20]. The point spread function (PSF) reconstruction as used in modern PET scanners not only improves sensitivity but it overestimates SUV_max_ [21]. The SUV_peak_ has been shown to provide a slightly more robust alternative for assessing the most metabolically active region of a tumor [22–25].

In a recent study of Ito et al., whole-body MTV obtained from baseline PET/CT scans has been shown to be a strong independent prognostic factor among other clinical prognostic factors in melanoma patients treated with ipilimumab [26]. Son et al. observed that among patients with primary cutaneous melanoma, both MTV and TLG are strong prognosticators of survival [27].

Melanoma patients with an elevated serum LDH level have a higher tumor ^18^F-FDG uptake, however, without full coincidence [8]. The prediction of patient prognosis and the assessment of early response to immunotherapy have become areas of intensive investigation, because unnecessary toxicities or aggressive treatments should be avoided [28].

In this study we investigated the association of tumor volumetric parameters in melanoma patients undergoing ^18^F-FDG-PET/CT with serologic tumor markers and inflammatory markers and the role as independent imaging predictors for overall survival.

## Methods

Ethics approval was obtained from the local ethics committee (Project number: 064/2013B01). Informed consent was obtained from all patients included in the study.

### Patient cohort

The underlying study population consisted of patients with advanced melanoma, who were enrolled in a local PET/CT registry between April 2013 and January 2015 [29, 30]. All patients were initially intended for radical metastasectomy based on conventional imaging prior to the PET/CT examination. According to the melanoma guideline, PET/CT imaging is routinely recommended for patients with stage III and IV melanoma and in case of high risk melanoma (ulceration or tumor thickness above 4 mm) or suspect findings in the follow-up (i.e. US or serologic tumor markers) in patients with stage I and II [31].

After having performed the PET/CT scan, patients were re-evaluated regarding the intended management plan (surgery, systemic therapy, watchful watching). The final treatment was a consensus decision of a tumor-board on the basis of the PET/CT result in agreement with the patients. In case of ^18^F-FDG avid metastases, a corresponding surgical or systemic therapy was initiated. If no vital metastases were confirmed, patients underwent watchful waiting.

### ^18^F-FDG-PET/CT imaging

All PET/CT examinations were performed on a state-of-the art clinical scanner (Biograph mCT®, Siemens Healthineers). All patients fasted overnight before examination. Approximately 300 MBq ^18^F-FDG were injected intravenously 60 min prior to image acquisition. Standardized CT examination protocols included weight-adapted 90–120 ml intravenous CT contrast agent (Ultravist 370®, Schering AG). Portal-venous phase acquisitions were obtained with 70s delay time using a tube voltage of 120 kV and a reference dose of 200mAs. Image reconstruction was performed using iterative CT reconstruction (Siemens SAFIRE®, Forchheim).

PET was acquired from the skull to the mid thigh level over six to eight bed positions and reconstructed using a 3D ordered subset expectation maximization algorithm (two iterations, 21 subsets, Gaussian filter 2.0 mm, matrix size 400 × 400, and slice thickness 2.0 mm). In case of known metastases at the extremities, PET acquisition was expanded accordingly. PET acquisition time was 2–3 min per bed position.

### Quantification of tumor lesion ^18^F-FDG uptake and serologic markers

Segmentation of metastatic tumor lesions was performed by two readers in consensus using approved software for quantification of PET parameters on Syngo.via VB 30A (Siemens Healthineers). Metastatic lesions included all lesions which were characterized by substantially increased ^18^F-FDG uptake. Segmentation of each lesion was manually performed using 50%-isocontour VOIs for quantification. Whole-body MTV and whole-body TLG were calculated as the sum of all quantified metastatic lesions per patient. The SUV_peak_ of the metastatic lesion with the highest ^18F^-FDG uptake in a patient was calculated using an automated computed maximal mean SUV in a 1.0-cm^3^ spherical volume within the tumor [24]. The documented patient’s SUV_peak_ is defined as the highest value derived from all lesions within a patient.

As part of the staging procedures in melanoma patients, serum LDH, serum S-100 protein and the acute-phase proteins CRP and AP were routinely determined by the in-house laboratory. Serologic tumor markers were extracted from the clinical data base within 45 days before up to 7 days after PET/CT and acute-phase-proteins 20 days before up to 7 days after PET/CT. The upper limits of the reference ranges were: 250 U/l for serum LDH, 0.1 μg/l for serum S-100 protein, 0.5 μg/dl for CRP and 130 U/l for AP.

### Data analysis

Statistical analyses and graphical illustrations were performed using SPSS Version 22 (IBM Corporation).

### Association of PET parameters and serologic markers

First, we checked for potential associations between the serum parameters and the whole-body MTV, whole-body TLG and SUV_peak_ by direct correlation of the absolute values. Second, we analyzed these associations separately in patients undergoing surgical or systemic treatment after PET/CT. Interactions between PET parameters and serologic markers were analyzed by bivariate correlation. Further, a multiple linear regression was calculated to predict the whole-body MTV, whole-body TLG and SUV_peak_ based on serologic markers. The strength of the linear relationships between the variables was measured by calculating the Pearson correlation coefficient which was denoted by r_P_. The predictive value of serum LDH for differentiation between patients with and without metastases and between patients with whole-body MTV, whole-body TLG and SUV_peak_ above or below the cohort’s median was assessed by computing a receiver operating characteristic (ROC) curve and by calculation of the area under the curve (AUC).

### Survival analysis

Overall patient survival recorded between date of PET/CT and death was assessed for all patients based on patient records. In a first step, we performed an univariate analysis to identify PET markers including whole-body MTV, whole-body TLG and SUV_peak_ as well as serologic parameters including LDH, serum-100, AP and CRP associated with OS. In a second step, the factors that were identified as being significant by univariate analysis (*p* <  0.05) were entered into a Cox multivariate regression analysis model. A forward stepwise multivariate regression analysis was carried out to identify the factors that remained significant after multivariate analysis. The variables with *p* <  0.05 were entered and those with *p* > 0.10 were removed.

Third, we compared the OS between patients with and without metastases on PET/CT, between patients with whole-body MTV, whole-body TLG and SUV_peak_ above or below the cohort’s median and between patients with normal or elevated serologic parameters (serum LDH, serum S-100 protein, CRP, AP). Fourth, OS was analyzed in the patient subgroups undergoing surgical or systemic treatment after PET/CT for normal and elevated PET parameters and serologic markers.

To analyze differences of overall survival between the groups, we performed Kaplan-Meier analyses. The differences between the Kaplan Meier survival curves were evaluated by non-parametric log-rank tests. Optimal thresholds were identified for each marker, which best separated the subgroups (lowest *p*-value from log-rank test). The significance level was set at a p-value of < 0.05. Estimated mean survival times were derived from Kaplan-Meier analyses.

## Results

### Study population

107 consecutive patients (52 female; mean age 65 ± 13.1 years) with malignant melanoma who were selected for potential surgical metastasectomy prior PET/CT were evaluated. Tumors were staged according to the eighth edition AJCC Cancer Staging Manual [32].

Five patients had stage I, three patients stage II, 42 patients stage III and 57 patients stage IV melanoma according to PET/CT. The eight early stage (I and II) patients had been scheduled for surgery for suspicious findings in CT or US. On the basis of clinical findings and PET/CT results, 52 patients (48.6%) were selected for surgical treatment whereas 32 patients (29.9%) were selected for systemic therapy. Two patients (1.9%) underwent palliative radiotherapy and one patient (0.9%) underwent isolated extremity perfusion. 20 patients (18.7%) underwent watchful waiting. Detailed patient characteristics are listed in Table 1.

**Table 1:** Patient characteristics

Characteristic	No. of patients	%
Sex	55	51.4
Male	52	48.6
Female		
Age, years		
Mean	65	
Interquartile range (25–75)	55–74	
Stage (AJCC 2009)		
Stage I	5	4.7
Stage II	3	2.8
Stage III	42	39.3
Stage IV	57	53.3
Histological melanoma type		
Superficial	35	32.7
Nodular	21	19.6
Lentigo maligna	7	6.5
Acral lentiginous	15	14.0
Mucosal	6	5.6
Other	23	21.4
Treatment after PET/CT		
Surgical	52	48.6
Systemic	32	29.9
Other	3	2.8
None	20	18.7

### Association of PET parameters and serologic markers

**Fig. 1:**
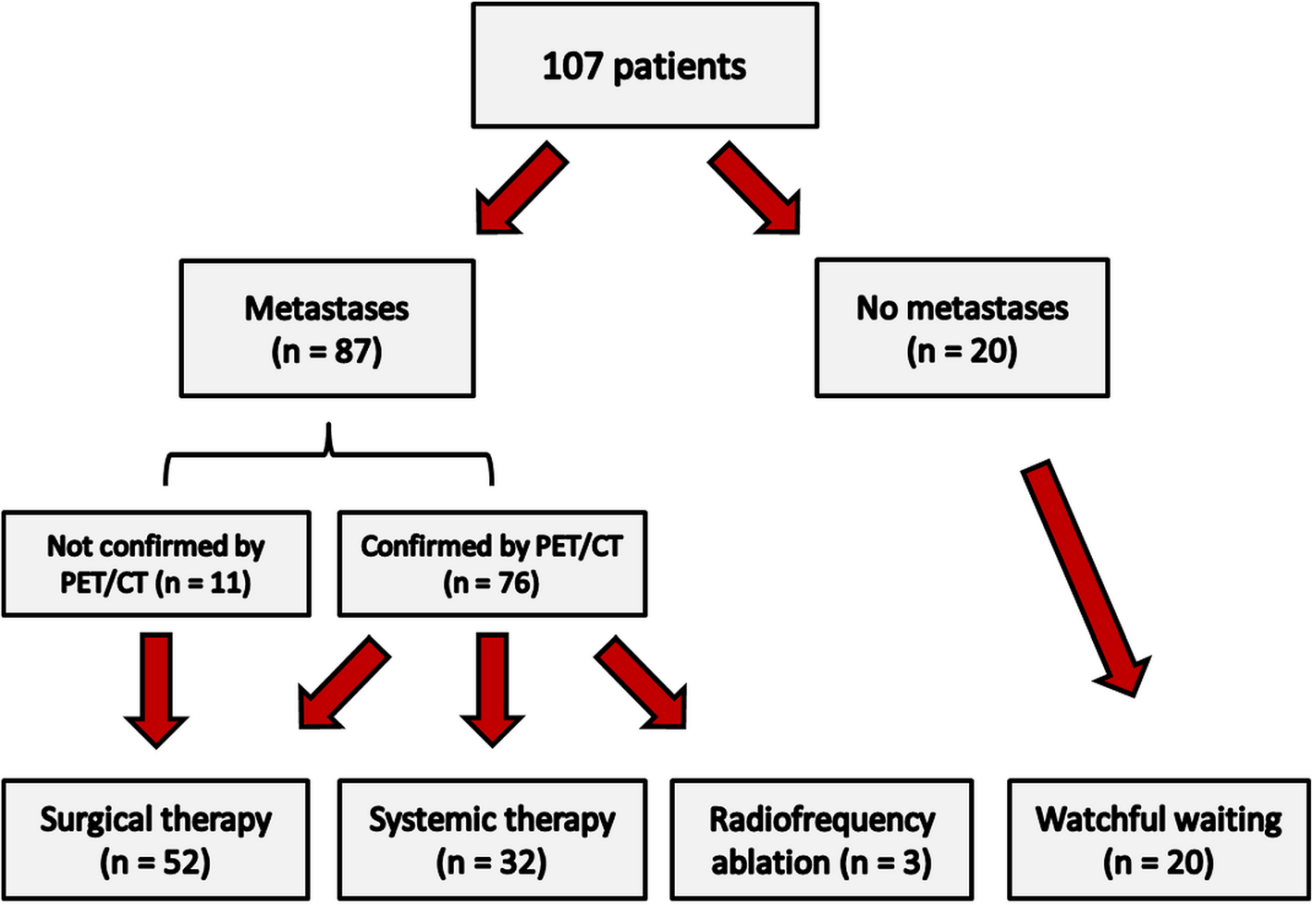
Patient flowchart

PET/CT findings and results of the laboratory are shown in Table 2. A total of 87/107 patients (81.3%) had histologically confirmed metastases. ^18^F-FDG avid lesions have been identified in 76/107 patients (71.0%) by PET/CT allowing for manual segmentation. 11/107 patients (10.3%) had small cutaneous in-transit metastases which were either not completely recorded by PET/CT scan or not clearly quantifiable (Fig. 1).

**Table 2:** PET/CT and laboratory findings

PET/CT and laboratory findings	No. of patients	%
Metastases	87	81.3
^18^F-FDG avid metastases quantifiable by PET/CT	76	71.0
Whole body metabolic tumor volume (MTV, cm^3^)		48.6
Median	2.74	
Interquartile range (25–75)	0.30–9.22	
Whole-body total lesion glycolysis (TLG)		
Median	13.0	
Interquartile range (25–75)	1.17–64.30	
SUV_peak_		
Median	6.7	
Interquartile range (25–75)	2.53–12.60	
Serum lactate dehydrogenase (mean: 241 [130–960] U/l)	84	78.5
Normal	64	76.2
Increased	18	21.4
Serum S-100 protein (mean: 0.14 [0.02–3.0] μg/l)	82	76.6
Normal	59	72.0
Increased	23	28.0
C-reactive protein (mean: 3.0 [0.01–16.7] mg/dl)	72	67.3
Normal	28	38.9
Increased	44	61.1
Alkaline phosphatase (mean: 91 [36–175] μg/l)	68	63.6
Normal	56	82.4
Increased	12	17.6

Current serologic parameters were available as follows:

- Serum LDH was available in 84/107 patients (78.5%), which could be correlated with PET parameters in 67 patients.
- Serum S-100 protein was available in 82/107 patients (76.6%), which could be correlated with PET parameters in 68 patients.
- CRP was available in 72/107 patients (67.3%), which could be correlated with PET parameters in 59 patients.
- AP was available in 68/107 patients (63.6%), which could be correlated with PET parameters in 60 patients.

**Fig. 2:**
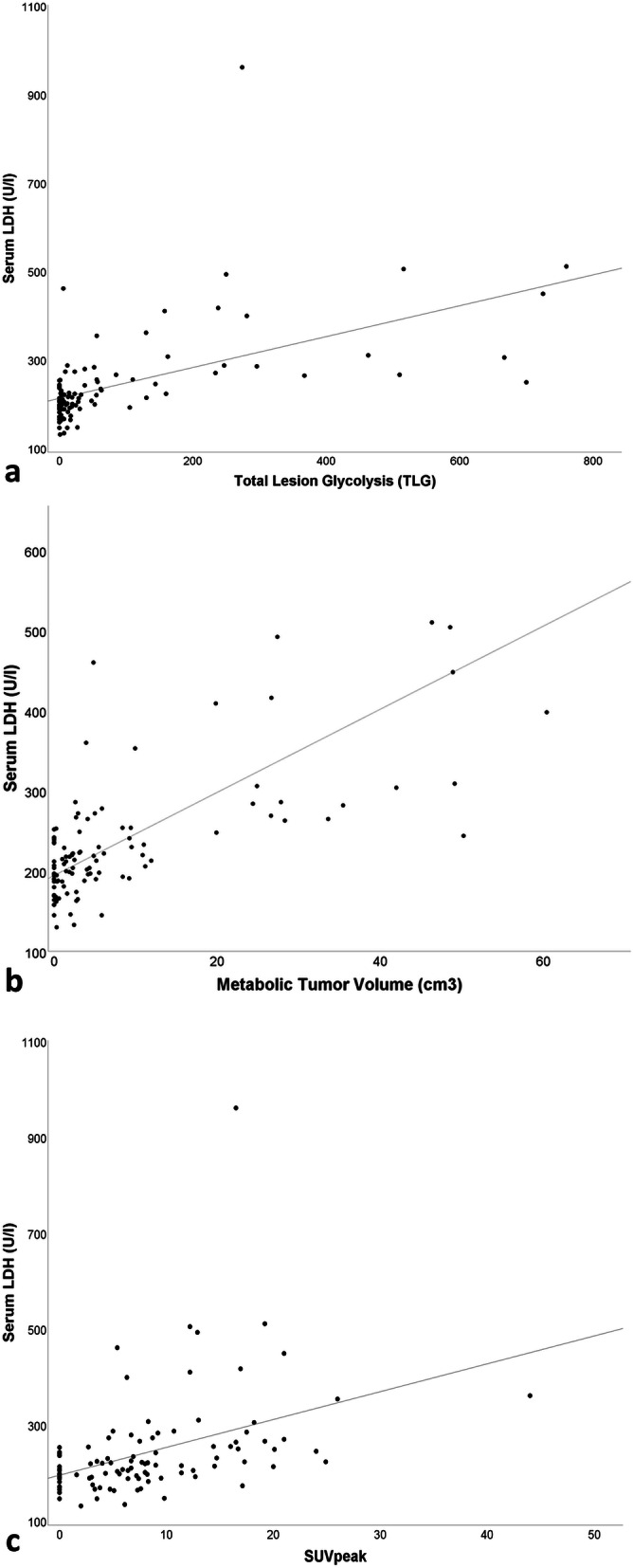
**a** Bivariate correlation curves between serum LDH and whole-body TLG (rP = 0.62, p <  0.001), between serum LDH and whole-body MTV (rP = 0.73, *p* <  0.001) (**b**) and between serum LDH and SUV_peak_ (rP = 0.55, p <  0.001) (**c**)

Serum LDH and serum S-100 protein showed a significantly positive correlation (r_P_ = 0.82, *p* <  0.001). In the whole patient cohort, serum LDH was strongly associated with whole-body MTV (r_P_ = 0.73, *p* <  0.001) and moderately associated with whole-body TLG (r_P_ = 0.62, *p* <  0.001) and SUV_peak_ (r_P_ = 0.55, p <  0.001) (Fig. 2). S-100 protein showed a moderate association with MTV (r_P_ = 0.54, *p* <  0.001) and TLG (r_P_ = 0.48, *p* <  0.001) and a weak association with SUVpeak (r_P_ = 0.42, p <  0.001). A strong association was observed between CRP and MTV (r_P_ = 0.66, p <  0.001) and a moderate to weak association between CRP and TLG (r_P_ = 0.53, p <  0.001) and CRP and SUV_peak_ (r_P_ = 0.45, *p* <  0.001). A weak association was also observed between AP and MTV (r_P_ = 0.39, *p* <  0.001) and AP and TLG (r_P_ = 0.29, *p* <  0.01). AP and SUV_peak_ were not associated (r_P_ = 0.16, *p* = 0.2).

The separate analysis for patients receiving surgical treatment after PET/CT revealed strong associations between serum LDH and MTV (r_P_ = 0.82, *p* <  0.001) and serum LDH and TLG (r_P_ = 0.74, *p* <  0.001) and between serum S-100 protein and MTV (r_P_ = 0.66, p <  0.001). Moderate associations were observed between S-100 protein and TLG (r_P_ = 0.60, *p* <  0.001), SUV_peak_ and serum LDH (r_P_ = 0.60, *p* <  0.001) and SUV_peak_ and S-100 protein (r_P_ = 0.48, *p* <  0.001).

In the subgroup of patients undergoing systemic treatment after PET/CT, MTV and serum LDH (r_P_ = 0.61, *p* <  0.001) and MTV and S-100 protein were moderately associated (r_P_ = 0.56, *p* <  0.001). Moderate associations were also observed between TLG and serum LDH (r_P_ = 0.47, *p* <  0.001), TLG and S-100 protein (r_P_ = 0.42, *p* <  0.001), SUV_peak_ and serum LDH (r_P_ = 0.51, *p* <  0.001) and SUV_peak_ and S-100 protein (r_P_ = 0.43, *p* <  0.001). The results of the bivariate correlation analyses are listed in Table 3.

**Table 3:** Bivariate correlation analysis between PET and serologic parameters

	MTV (cm3)		TLG		SUV_peak_	
All patients (*n* = 107)	r_P_	*p*-value	r_P_	*p*-value	r_P_	*p*-value
Serum LDH (U/l)	0.73	< 0.001	0.62	< 0.001	0.55	< 0.001
Serum S-100 protein (μg/l)	0.54	< 0.001	0.48	< 0.001	0.42	< 0.001
C-reactive protein (mg/dl)	0.66	< 0.001	0.53	< 0.001	0.45	< 0.001
Alkaline phosphatase (μg/l)	0.39	< 0.001	0.29	< 0.01	0.16	0.2
Surgical treatment (*n* = 52)						
Serum LDH (U/l)	0.82	< 0.001	0.74	< 0.001	0.60	< 0.001
Serum S-100 protein (μg/l)	0.66	< 0.001	0.60	< 0.001	0.48	< 0.001
C-reactive protein (mg/dl)	0.62	< 0.001	0.50	< 0.001	0.13	0.4
Alkaline phosphatase (μg/l)	0.44	0.007	0.21	0.2	0.12	0.1
Systemic treatment (*n* = 32)						
Serum LDH (U/l)	0.61	< 0.001	0.47	< 0.001	0.51	< 0.001
Serum S-100 protein (μg/l)	0.56	< 0.001	0.42	< 0.001	0.43	< 0.001
C-reactive protein (mg/dl)	0.41	0.06	0.31	0.2	0.48	0.02
Alkaline phosphatase (μg/l)	0.34	0.06	0.37	0.04	0.21	0.3

The ROC analysis for differentiation between patients with and without metastases revealed a cut-off value of 198 U/l for serum LDH with an AUC of 0.81 (sensitivity 0.80; specificity 0.72). A significant regression equation was found: F (4,52) = 26.9, *p* <  0.0001, with R^2^ of 0.67. Both serum LDH and CRP were significant predictors of whole-body MTV. Whole-body MTV increased 0.84 cm^3^ for each U/l serum LDH and 1.83 cm^3^ for each mg/dl CRP.

### Overall survival

At the time of analysis in February 2020, 55/107 patients (51.4%) had died, whereas 47/107 patients (43.9%) were still alive. In 5/107 patients (4.7%) survival data were not available.

Univariate analysis revealed that whole-body MTV (< 2.74cm^3^ vs. > 2.74cm^3^), whole-body TLG (< 13.0 vs. > 13.0), SUV_peak_ (< 6.7 vs. > 6.7), as well as the serologic parameters LDH (normal vs. increased), and S-100 protein (normal vs. increased) were significant predictors of overall survival. Multivariate analysis for overall survival including the significant parameters revealed that whole-body TLG greater than 13 (hazard ratio [HR], 3.30; 95% CI, 1.6–6.80, *p* = 0.001), and whole-body MTV greater than 2.74 cm^3^ (HR, 2.29; 95% CI, 1.12–4.70, *p* = 0.02) remained independent prognostic factors (Table 4).

**Table 4:** Univariate and multivariate survival analyses of PET and serologic parameters

		Univariate analysis			Multivariate analysis		
		Hazard ratio	95% CI	*p*-value	Hazard ratio	95% CI	*p*-value
PET parameters							
Whole-body MTV	< 2.74 cm3	1.00	–	–	–	–	–
	> 2.74 cm3	4.90	1.93–12.46	***0.001***	2.29	1.12–4.70	***0.02***
Whole-body TLG	< 13.0	1.00	–	–	–	–	–
	> 13.0	3.86	1.98–7.55	***< 0.0001***	3.30	1.60–6.80	***0.001***
SUVpeak	< 6.7	1.00	–	–	–	–	–
	> 6.7	2.81	1.48–5.33	***0.002***	2.29	1.16–4.52	*0.02*
Serologic parameters							
LDH	normal	1.00	–	–	–	–	–
	increased	2.18	1.17–4.05	***0.01***	1.18	0.53–2.63	0.68
S-100 protein	normal	1.00	–	–	–	–	–
	increased	2.09	1.14–3.82	***0.02***	1.33	0.63–2.81	0.46
Alkaline phosphatase	normal	1.00	–	–	–	–	–
	increased	1.32	0.69–2.52	0.40	–	–	–
C-reactive protein	normal	1.00	–	–			
	increased	0.89	0.44–1.81	0.74			

**Fig. 3:**
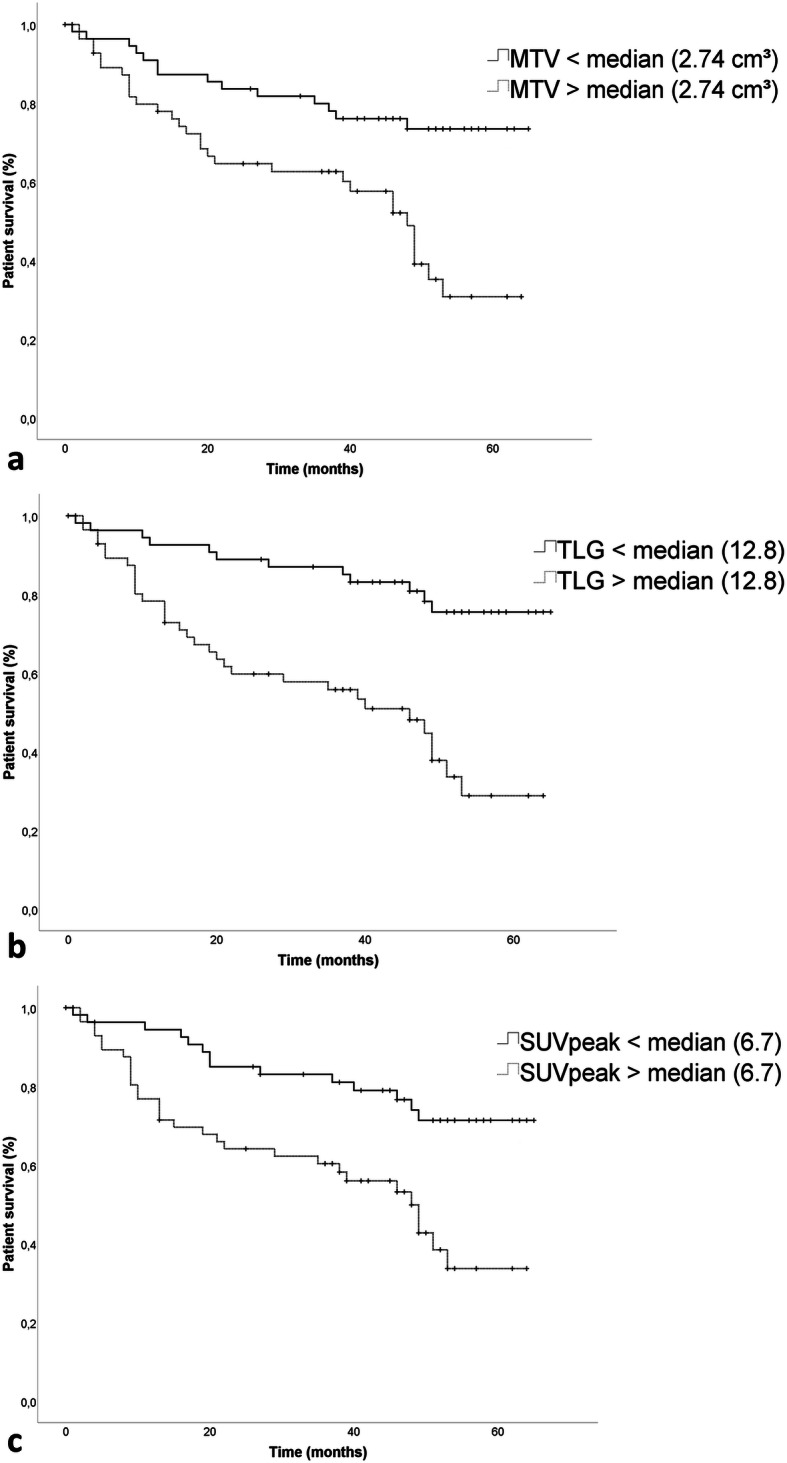
**a** Estimated overall survival in patients with MTV above the cohort’s median (42.8 ± 3.3 months) compared to patients with MTV below the cohort’s median (51.2 ± 2.7 months, p <  0.001), (**b**) in patients with TLG above the cohort’s median (37.1 ± 3.2 months) compared to patients with TLG below the cohort’s median (55.9 ± 2.5 months, *p* <  0.001) and (**c**) in patients with SUV_peak_ above the cohort’s median (39.9 ± 3.2 months) compared to patients with SUV_peak_ below the cohort’s median (54.1 ± 2.7 months, p <  0.001)

Patients with ^18^F-FDG avid metastases had a significantly reduced estimated OS (43.1 ± 2.7 months) compared to patients without ^18^F-FDG avid metastases (55.7 ± 2.8 months, *p* <  0.01) (Fig. 3a). Patients with whole-body MTV, whole-body TLG or SUV_peak_ above the cohort’s median had a significantly (*p* <  0.001) reduced estimated OS compared to patients with corresponding PET parameters below cohort’s median (MTV: 42.8 ± 3.3 months vs. 51.2 ± 2.7 months; TLG: 37.1 ± 3.2 months vs. 55.9 ± 2.5 months; SUVpeak: 39.9 ± 3.2 months vs. 54.1 ± 2.7 months) (Fig. 3).

**Fig. 4:**
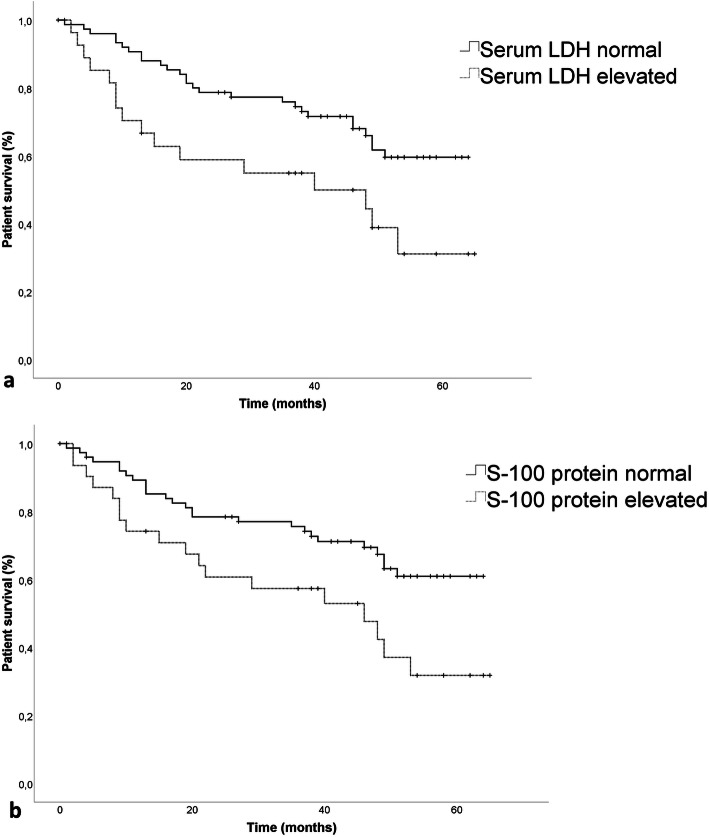
(**a**) Estimated overall survival in patients with elevated serum LDH (36.5 ± 4.9 months) compared to patients with normal serum LDH (49.2 ± 2.4 months, *p* = 0.01) and (**b**) in patients with elevated serum S-100 protein (37.9 ± 4.4 months) compared to patients with normal serum S-100 protein (49.0 ± 2.5 months, *p* = 0.01)

Correspondingly, an elevated serum LDH was accompanied with a significantly lower OS (36.5 ± 4.9 months) compared to patients with normal serum LDH (49.2 ± 2.4 months, *p* = 0.01), which was also observed in patients with an elevated serum S-100 protein (37.9 ± 4.4 months) compared to patients with a normal serum S-100 protein (49.0 ± 2.5 months, p = 0.01) (Fig. 4). No differences in OS could be observed between patients with an elevated (43.3 ± 4.4 months) or normal AP (45.3 ± 3.2 months, *p* = 0.48) and an elevated (47.8 ± 3.5 months) or normal CRP (41.9 ± 4.4 months, *p* = 0.41).

In the subgroup of patients undergoing surgical treatment after PET/CT, OS was significantly reduced in case of ^18^F-FDG avid metastases (*n* = 41, 51.7 ± 4.0 months) compared to patients without ^18^F-FDG avid metastases (*n* = 11, 60.9 ± 7.2 months, *p* <  0.05). This observation was similar to patients with elevated serum S-100 protein (41.8 ± 5.9 months) compared to patients with normal serum S-100 protein (50.2 ± 3.4 months), however, without statistical significance (*p* = 0.07).

## Discussion

In this study, we investigated the clinical and prognostic value of volumetric PET parameters in a patient cohort with advanced melanoma undergoing ^18^F-FDG-PET/CT by direct correlation with the established serologic tumor markers LDH and S-100 protein and the inflammatory markers AP and CRP.

A strong association was observed between the whole-body MTV and LDH, whereas whole-body TLG was moderately associated with LDH. Moderate associations were also detected between LDH and SUV_peak_ and between S-100 protein and both MTV and TLG. Similar associations were observed in the patient subgroups who underwent surgical or systemic treatment after PET/CT.

To the best of our knowledge, there are no reports so far reporting on direct correlations of volumetric imaging markers and the established serologic tumor markers LDH and S-100 protein in melanoma. A possible explanation for the strong association between MTV, TLG and LDH is that the conversion of pyruvate to lactate by LDH produces NAD^+^, and the NADH/NAD^+^ ratio is thought to be important in various oxidoreductase-based metabolic reactions which are upregulated in melanoma cells [33]. Increasing serum values of LDH are correlated with tumor progression and are therefore found in higher tumor stages [13]. However, it has been shown that LDH is less sensitive in early disease stages and as a predictor of metastatic relapse [34, 35].

The volumetric parameters MTV/TLG and the SUV_peak_ showed a stronger association with serum LDH as with CRP. This is in concordance with the study results of de Heer et al. showing significantly higher MTV, TLG and SUV_peak_ in melanoma patients with elevated LDH [8]. CRP is synthetized in response to cytokines such as interleukin-6 (IL-6), which is produced by melanoma cells [36]. However, CRP might also be synthetized by activated T cells, macrophages or monocytes which are also responsible for elevated IL-6 levels in response to inflammation [37]. Therefore, CRP is not a marker which is exclusively increased in melanoma.

We observed a strong association between the serum markers LDH and S-100 protein. Therefore, it is not surprising, that S-100 protein was also associated with MTV, TLG and SUV_peak_. It has been observed that S-100 protein has a prometastatic attribute in melanoma by influencing cell growth and differentiation and interaction with coexpressed receptor for advanced glycation endproducts (RAGE) [38, 39]. It has been shown that serum concentrations of S-100 correlate with clinical melanoma stage [13]. In asymptomatic melanoma patients, S-100 protein has been proven to be a useful tool for discovering tumor progression which could be confirmed by PET/CT [40]. However, abnormal elevated S-100 levels may attributed to other causes such as inflammatory and infectious diseases [41–43].

Our survival analysis revealed that both whole-body MTV and whole-body TLG are independent prognostic factors. Patients with ^18^F-FDG avid metastases or MTV/TLG and SUVpeak above the cohort’s median had a significantly reduced survival, which was similarly observed in patients with an elevated serum LDH or elevated serum S-100 protein. Patients undergoing surgical treatment after PET/CT had a reduced OS in case of ^18^F-FDG avid metastases, which was similarly observed in patients with elevated serum S-100 protein above the cohort’ median.

This is in concordance with other studies demonstrating that both whole-body MTV, whole-body TLG, SUV_peak_ and serum LDH are independent prognostic factors in patients with malignant melanoma [8, 26, 27]. Ito et al. combined information about PET parameters and clinical factors showing that melanoma patients with high serum LDH in combination with elevated whole-body MTV had a worse prognosis than patients with a high serum LDH or an elevated MTV alone [25]. Differences in survival between patients with a sum of SUV_peak_ above and below the cohort’s median were not significant, however, a trend was noted. In their study, patients were divided into subgroups with increased or not increased LDH referring to the upper limit of the normal range. We could additionally show that serum LDH and PET parameters are directly associated which may help clinicians to early identify melanoma patients who would particularly benefit from PET/CT imaging for staging. Further, Ito et al. included only patients with unresectable melanoma who were planned for ipilimumab immunotherapy and a part of their patient cohort had already undergone previous systemic therapy [44]. Son et al. evaluated the prognostic relevance of MTV, TLG and SUV_max_ in patients with primary cutaneous malignant melanoma [27]. The volumetric parameters MTV and TLG were significant prognostic factors for melanoma-specific survival, whereas SUV_max_ was not a significant factor [27]. Tumor volumetric parameters assessed on baseline PET/CT have been proven to be of prognostic value in various malignancies, including non-small cell lung cancer [45], lymphoma [46], breast cancer [47], head and neck cancer [48], and pancreatic cancer [20]. The clinical applicability of standard uptake value (SUV) for prognostic purposes in melanoma patients is still under discussion [49, 50]. The intra- and inter-patient heterogeneity in tumor lesion ^18^F-FDG uptake (SUV) among metastatic melanoma patients are major limitations [8].

S-100 protein and LDH have been reported as early prognostic markers for response and overall survival in melanoma patients treated with anti-PD-1 or combined anti-PD-1 plus anti-CTLA-4 antibodies [51]. Our observation that increased MTV is directly associated with serum LDH and S-100 in patients being accompanied with worse prognosis might therefore be a help for clinicians to early identify patients with an increased risk of relapse and which deserve particularly close monitoring. Perhaps, these would also be the patients who particularly benefited from a neoadjuvant therapy approach. No differences in OS could be observed between patients with elevated or normal acute-phase proteins (AP and CRP). An explanation is the low specificity of CRP and AP which may be elevated due to other reasons, for instance, inflammatory disorders [52, 53].

In our study, a cut-off value of 198 U/l for serum LDH could be defined which best differentiates between patients with or without ^18^F-FDG avid metastases (sensitivity 0.80; specificity 0.72). If the serum LDH rises above this cut-off value, vital tumor burden can be reasonably assumed. This finding is of great diagnostic relevance as an increasing serum LDH above this cut-off value may influence patient prognosis and it is below the generally accepted cut-off of 250 U/l. The early decision to perform a PET/CT in a clinical diagnostic setting should be considered.

Our study has limitations. Due to the retrospective design, a selection bias cannot be excluded. In addition, serologic parameters and survival data were not available for all patients. Volumetric parameters such as the MTV require an accurate lesion segmentation using a standardized segmentation method which has still not been established across clinical institutions. As all patients of our study cohort were examined at our institution, MTV was measured using the same segmentation method which includes a fixed relative threshold for all lesions. Further, we used a 50% threshold for the isocontour VOIs instead of the EANM recommended 41% threshold, which may underestimate volumetric PET parameters [54]. The rationale for this choice was that our mCT system uses PSF modeling, producing higher values of SUV compared to standard OSEM. Our data are the first to demonstrate that there is an association between the absolute values of PET parameters and established serologic tumor markers in melanoma patients. Further prospective studies with more patients and consideration of neoadjuvant therapy approaches should be conducted.

## Conclusions

Tumor volumetric parameters in ^18^F-FDG-PET/CT serve as prognostic imaging biomarkers in patients with advanced melanoma which are associated with established serologic tumor markers and inflammatory markers.

## Data Availability

The datasets generated and analyzed during the current study are not publicly available due to sensitive information but are available in anonymous form from the corresponding author on reasonable request.

## Abbreviations

AP: Alkaline phosphatase
AUC: Area under the curve
AJCC: American Joint Committee on Cancer
CTLA-4: T-lymphocyte-associated protein 4
CRP: C-reactive protein
EANM: European Association of Nuclear Medicine
^18^F-FDG: [18F]fluoro-2-deoxy-D-glucose
LDH: Serum lactate dehydrogenase
MBq: Megabecquerel
MTV: Metabolic tumor volume
OS: Overall survival
PET/CT: Positron emission tomography/computed tomography
PD-1: Programmed cell death protein 1
PSF: point spread function
RAGE: Receptor for advanced glycation endproducts
ROC: Receiver operating characteristic
SUV: Standardized uptake value
VOI: Volume of Interest
